# Supplementary material for: Efficient Bioprocess for Mixed PET Waste Depolymerization Using Crude Cutinase
Source: Polymers (Basel). 2025 Mar 14;17(6):763. doi: 10.3390/polym17060763 (PMC11946107; doi:10.3390/polym17060763)
Supplement: Supplementary file 1 [file polymers-17-00763-s001.zip › polymers-3496310-supplementary.pdf]

Title: **Efficient bioprocess for mixed PET waste depolymerization using crude cutinase**

Authors: Virender Kumar, Reinhard Wimmer, Cristiano Varrone*

Department of Chemistry and Bioscience, Aalborg University, Fredrik Bajers Vej 7H, 9220 Aalborg, Denmark

*Corresponding author:

Email: cva@bio.aau.dk (C. Varrone)

Supplementary Information

S1.1. Calculations for mass balance and molar yield (%)

The complete hydrolysis of 100 g PET (molar mass of repeating unit $C_{10}H_8 0_4$ =192.16 g/mol) would yield 0.5204 mol of TPA and EG.

Moles of PET =100 g/192.16g/mol =0.5204 mol

Theoretical Mass of TPA =0.5204 mol*166.13 g/mol =86.45 g

Theoretical Mass of EG=0.5204 mol*62.07 g/mol =32.29 g

Molar yield (%) $= \frac{\text{Tolal yield obtained in actual hydrolysis}}{\text{Theoretical molar yield}} X100$ (Eq. A1)

S1.2. Calculations for polymer weight loss

The weight loss of the PET film and powder was measured. For this, the weight of the empty reaction tube and then the weight after the addition of the polymer was measured. After the enzymatic treatment, the reaction tubes were centrifuged at 10000 rpm for 10 min. The supernatant was transferred to a new vial. The residual film or powder was washed with water twice carefully. After washing, the tubes were lyophilized for 24 h at 0.1 bar. The weight of the tube was measured, and the polymer weight loss was calculated using the following formula:

$\text{Weight loss of PET } (\%) = \frac{(\text{Initial weight} - \text{Final weight})}{\text{Initial weight}} \times 100$ (Eq. 2)

Table S1. Purification table of the enzymes produced in 1L of culture medium

	FastPETase (cytoplasmic, mg L^{-1})	LCC (Extracellular supernatant, mg L^{-1})	LCC^{ICCG} (Extracellular supernatant, mg L^{-1})
Total protein	322±	70±	75±

Purified (HisTrap FF)	26±	35±	32±
Specific activity (U/mg)*	968±15	1430±27	2187±19

***Specific activity calculated using pNPB assay**

Table S2. DSC analysis of different PET materials used in the study

Sample code	Sample name	$\Delta H_{c\ melt}$ (J/g)	$\Delta H_{c\ heat}$ (J/g)	ΔH_f (J/g)	ΔX_c (%)
F250A	PET 0.25 mm Amorphous	25.7	26.5	140.1	0.6
F250C	PET (0.25 mm) crystalline	9.0	58.2	140.1	35.1
P80A	PET powder cryo-milled (80 µm)	33.32	46.48	140.1	9.3
P750C	PET powder (50 µm)	2.5	55.4	140.1	37.7
P250C	PET powder (100 µm)	2.8	55.0	140.1	37.2
P250C	PET powder (250 µm)	1.50	61.3	140.1	42.6
P750A	PET powder (750 µm)	16.2	39.5	140.1	16.6
	PET tray (<500 µm)	19.8	42.1	140.1	15.9
	PET bottle (<500 µm)	9.5	48.21	140.1	28

Table S3. Composition of Minimal salt medium (M9)

**Chemical**	**g L^{-1}**
KH_2PO_4	15

$(NH_4)_2SO4$	1
KH_2PO_4	3
NaCl	5
$ZnCl_2$	0,1
$FeSO_4 \cdot 7H_2O$	0,15
$CaCl_2 \cdot 2H_2O$	0,02
**Followings autoclaved separately**	
Glycerol	5
MgSO4*7H2O	3
Mineral salt solution (composition below)	3 mL L^{-1}
$Al_2(SO_4)_3 \cdot 18H_2O$	2
$CoSO_4 \cdot 6H_2O$	0,75
$CuSO_4 \cdot 5H_2O$	2,5
H_3BO_3	0,5
$MnSO_4 \cdot H_2O$	24
$Na_2MoO_4 \cdot 2H_2O$	3
$NiSO_4 \cdot 3H_2O$	2,5
$ZnSO_4 \cdot 7H_2O$	15

Table S4. Different studies on enzyme production and PET degradation (Bioreactor scale)

Enzyme used	Organisms	Scale (L)	Growth Medium	Crude/purified	Type of waste	Enzyme loading (mg g^{-1}PET)	Substrate loading (g L^{-1})	Depolymerization (%)	Reference
LCC $ICCG^{DAQI}$	*E. coli*	2	LB/Autoinduction	Crude	PET fibers	3	10	97.5	[1]
LCC^{ICCG}	*E. coli*	1	ZYM-5052 auto-	Purified	Recycled PET	0.29	200	80	[2]

			induction medium						
LCC ICCG	*E. coli*	0.25	ZYM-5052 auto-induction medium	Purified	Postconsumer PET waste	1	165	98	[3]
FastPETase	*E. coli*	0.25	ZYM-5052 auto-induction medium	Purified	Postconsumer PET waste	1	165	20	[3]
HotPETase	*E. coli*	0.25	ZYM-5052 auto-induction medium	Purified	Postconsumer PET waste	1	165	26	[3]
PES-H1	*E. coli*	0.25	ZYM-5052 auto-induction medium	Purified	Postconsumer PET waste	1	165	80	[3]
FastPETase	*Pichia Pastoris*	10	BMGY	Crude	PET	2	5	90	[4]
LCC ICCG	*E. coli*	2	Minimal medium	Crude	Post-consumer PET trays	0.8	100	80	This work
LCC ICCG	*E. coli*	2	Minimal medium	Crude	Post-consumer PET trays and bottles	0.8	100	50	This work

Fig. S1 SDS PAGE analysis of LCC, and LCC^{ICCG}. Lanes contain different samples and are named as follows: Blank (B), purified LCC (L1), ICCG crude (I1 and I2), ICCG purified and concentrated (I3, I4), and protein marker (M). The proteins appear as a single band (LCC 29.9 kDa) with an actual molecular weight of 29.0 kDa, and (LCC^{ICCG} 27.2 kDa) with an actual molecular weight of 28.6 kDa. The gels were prepared twice with the gel shown representative of the set.

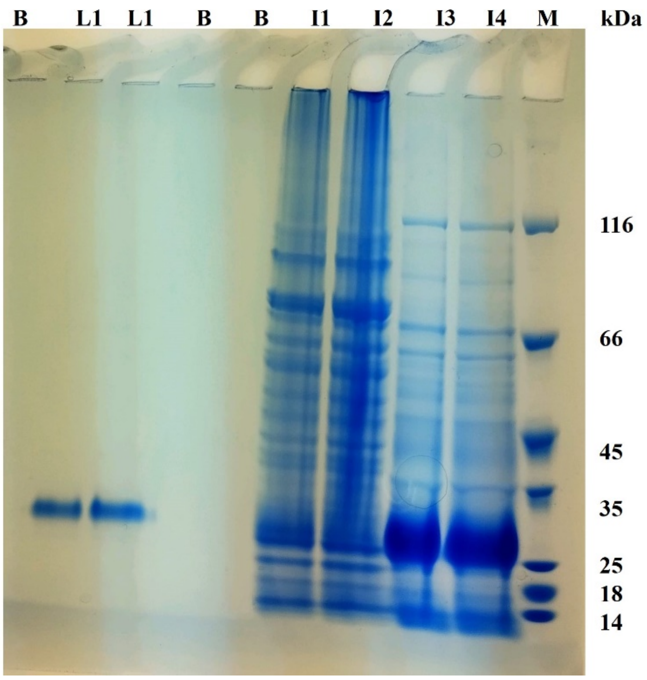

Fig. S2. Depolymerization (MHET and TPA release) of PET film (F250A) by (a) FastPETase, (b) LCC, and (c) LCC^{ICCG} in different buffers. The reaction mixture contained 50 mM of each buffer with different pH, 13 g L^{-1} PET film at 0.5 mg g^{-1} PET enzyme loading. It was incubated for 96 h at the respective optimum temperature of the enzymes i.e., 50 °C (FastPETase), 65 °C (LCC), and LCC^{ICCG} (72 °C) without any pH control.

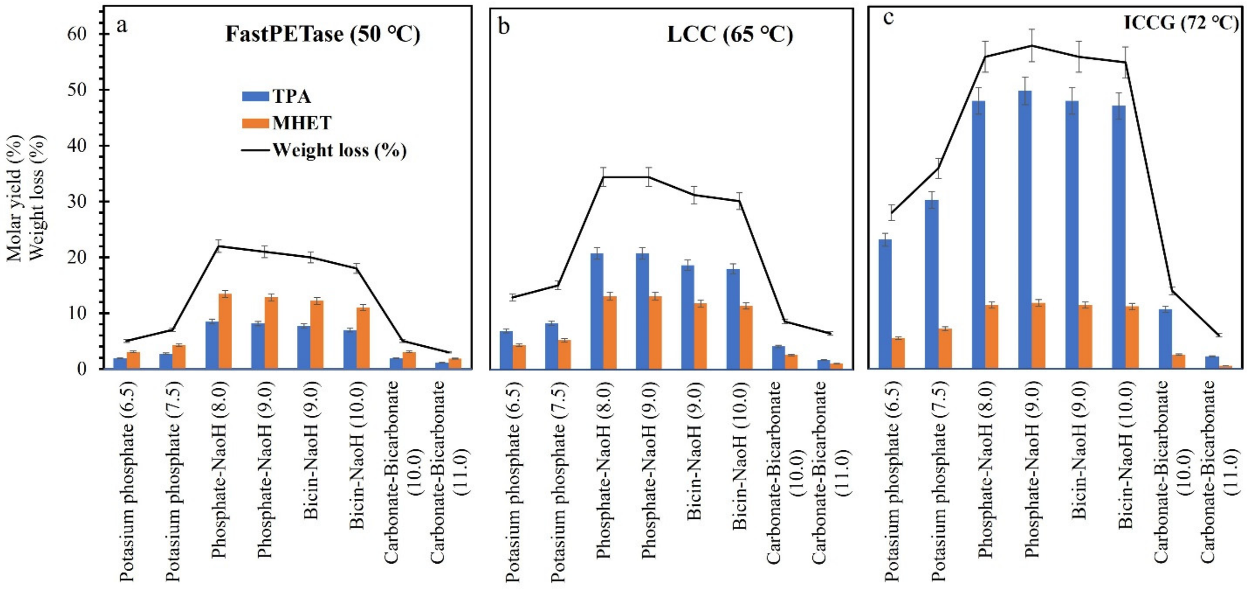

Fig. S3. Depolymerization (Weight loss and monomer release) of PET film (F250A) by (a) FastPETase, (b) LCC, and (c) LCC^{ICCG}. The reaction mixture contained 50 mM KH_2PO_4-NaOH buffer (pH 8.0), 13 g L^{-1} PET film at 0.5, 1 mg g^{-1} PET enzyme loading respectively at different temperatures (30, 40, 50, 65, 72, 80°C) without any pH control and incubated for 96 h.

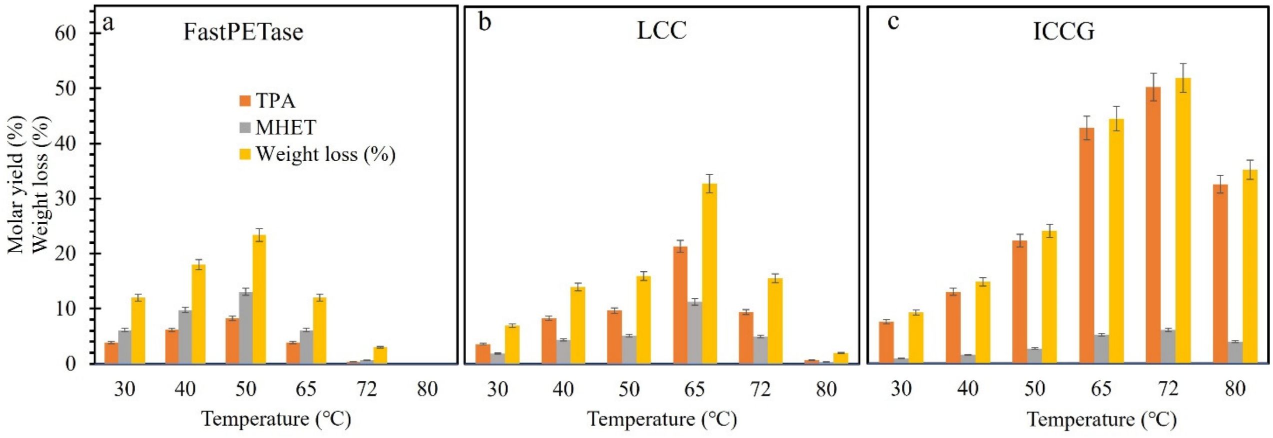

Fig. S4. Thermostability analysis of FastPETase, LCC, and LCC^{ICCG} at 50, 65, and 72℃. For the stability analysis, 2 µM enzyme was incubated at the respective temperatures for 36 h, and the residual activity was measured after different time intervals using pNPB assay in 50 mM phosphate-NaOH buffer (pH 8.0).

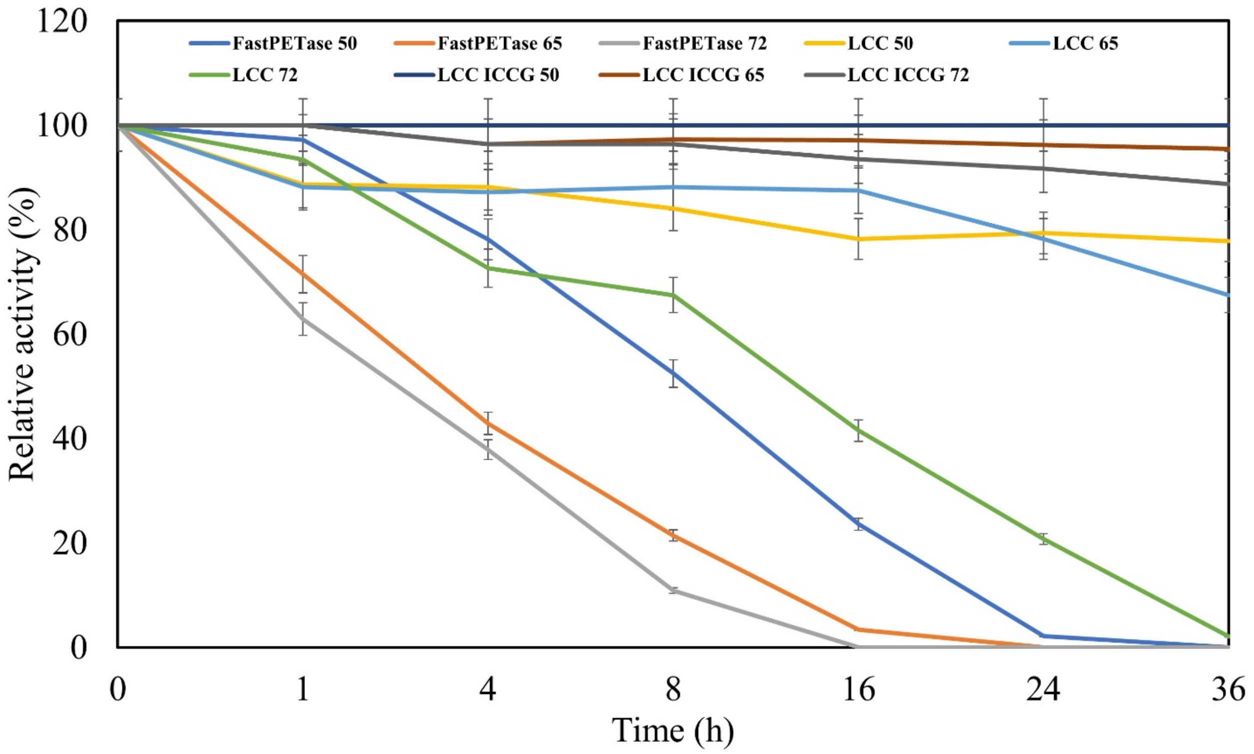

Fig. S5. ^{1}H-NMR (quantitative) spectra of supernatant after treatment of PET film with crude supernatant. The concentration of different compounds analyzed in the sample is presented in the tabular form.

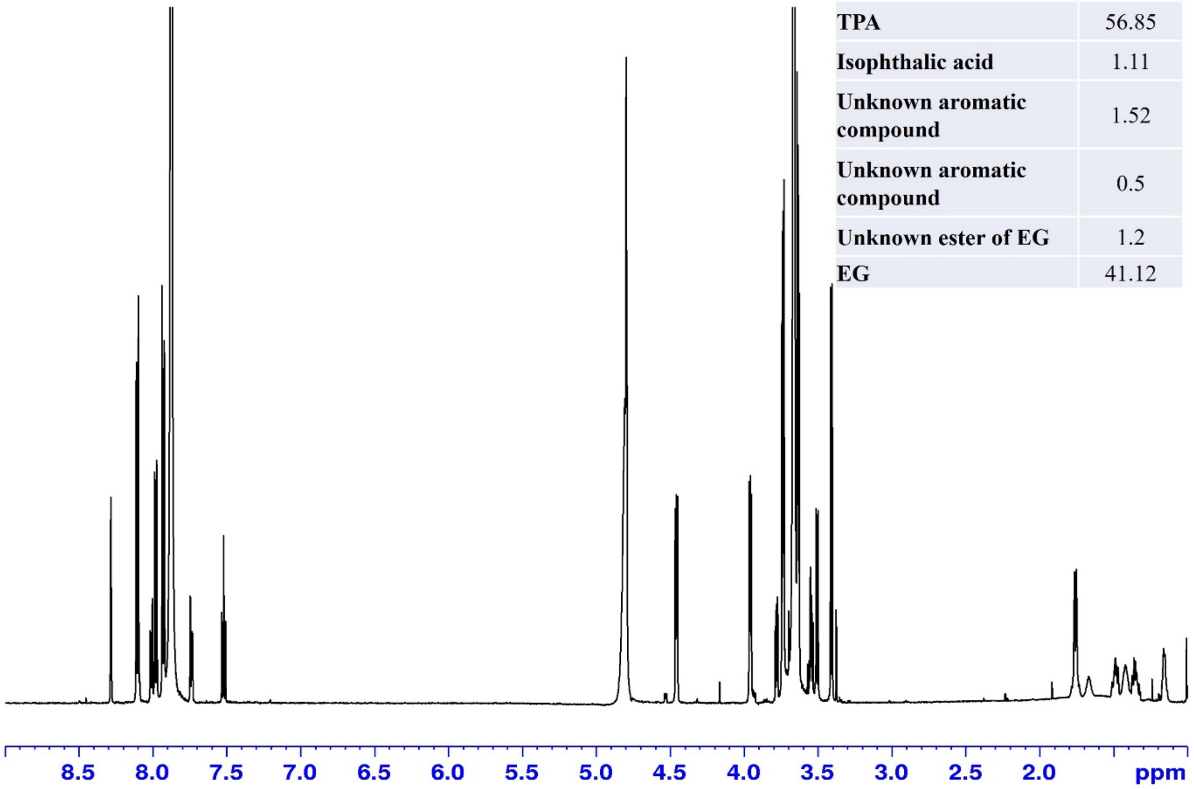

Compound	c [mM]
TPA	56.85
Isophthalic acid	1.11
Unknown aromatic compound	1.52
Unknown aromatic compound	0.5
Unknown ester of EG	1.2
EG	41.12

Fig. S6. ^{1}H-NMR (quantitative) spectra of supernatant after treatment of PET film with purified enzyme. The concentration of different compounds analyzed in the sample is presented in tabular form. The large peaks between 3.5.3.8 ppm belong to glycerol, which is a part of the concentrated protein solution.

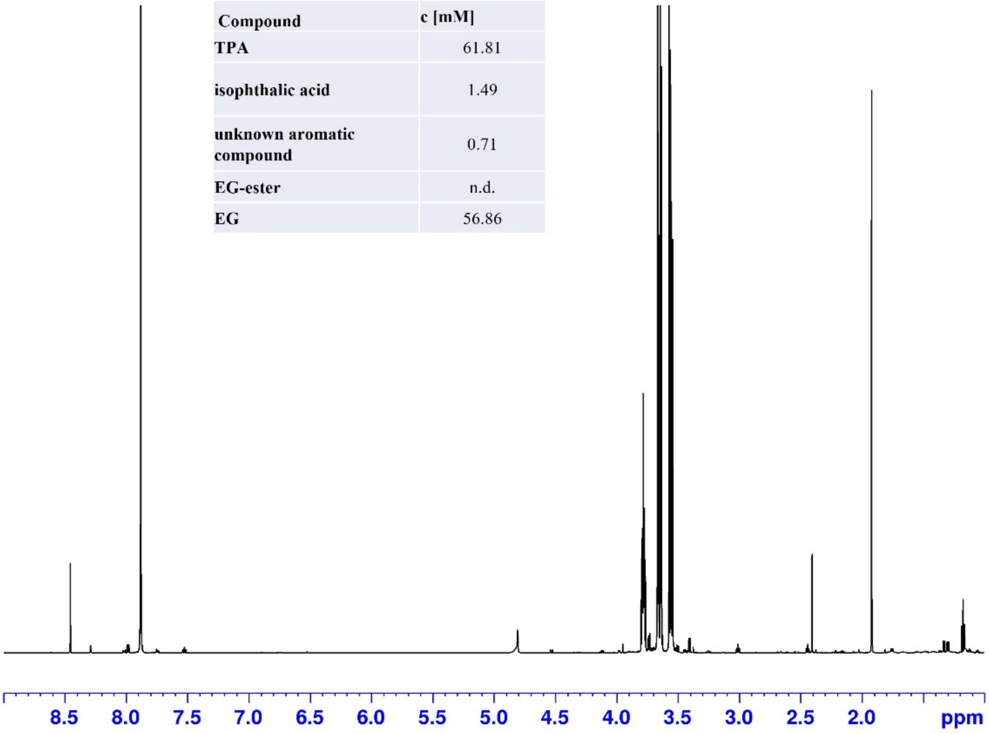

Compound	c [mM]
TPA	61.81
isophthalic acid	1.49
unknown aromatic compound	0.71
EG-ester	n.d.
EG	56.86

Fig. S7 1H-NMR of redissolved TPA in water at pH 7.85. Apart from TPA, the sample also contains isophthalic acid, glycerol, and ethylene glycol. The assignment of NMR resonances is given.

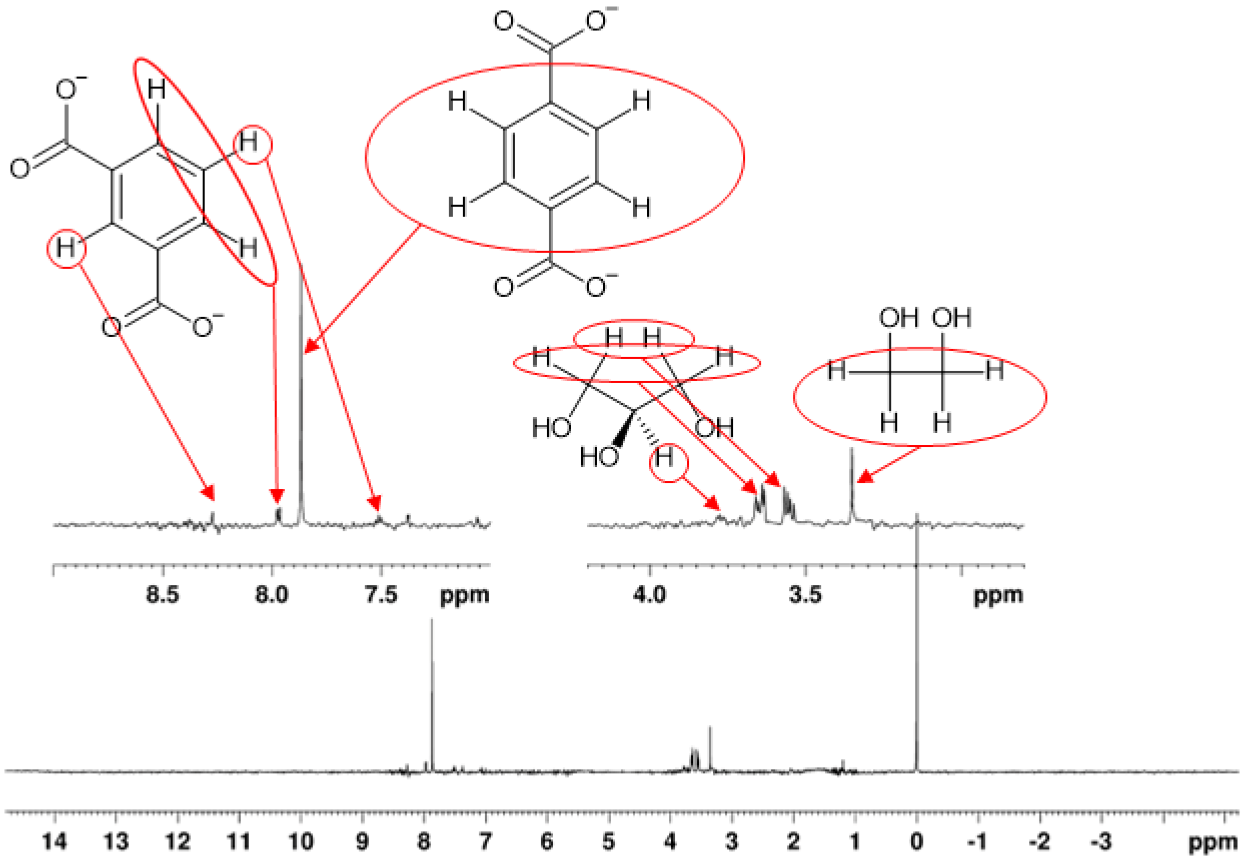

Fig. S8. Mixed PET waste used in the present study

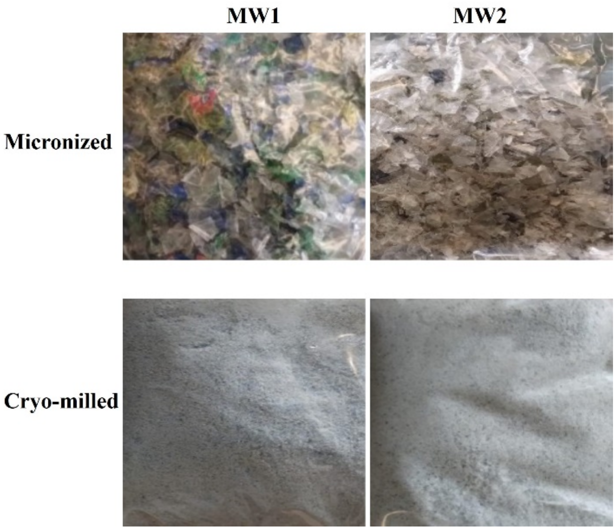

Fig. S9. ^{1}H-NMR (quantitative) spectra of supernatant after treatment of MW1 with crude supernatant.

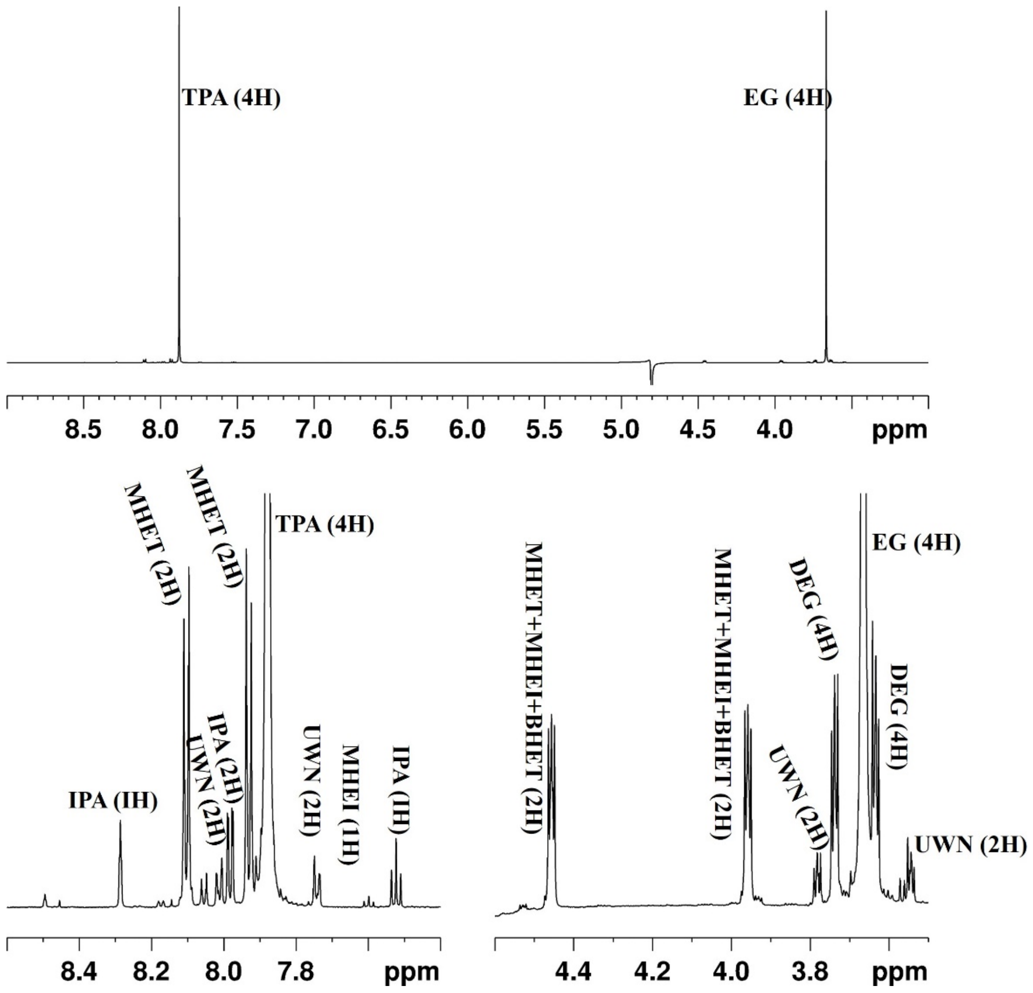

Fig. S10. ^{1}H-NMR (quantitative) spectra of supernatant after treatment of MW2 with crude supernatant.

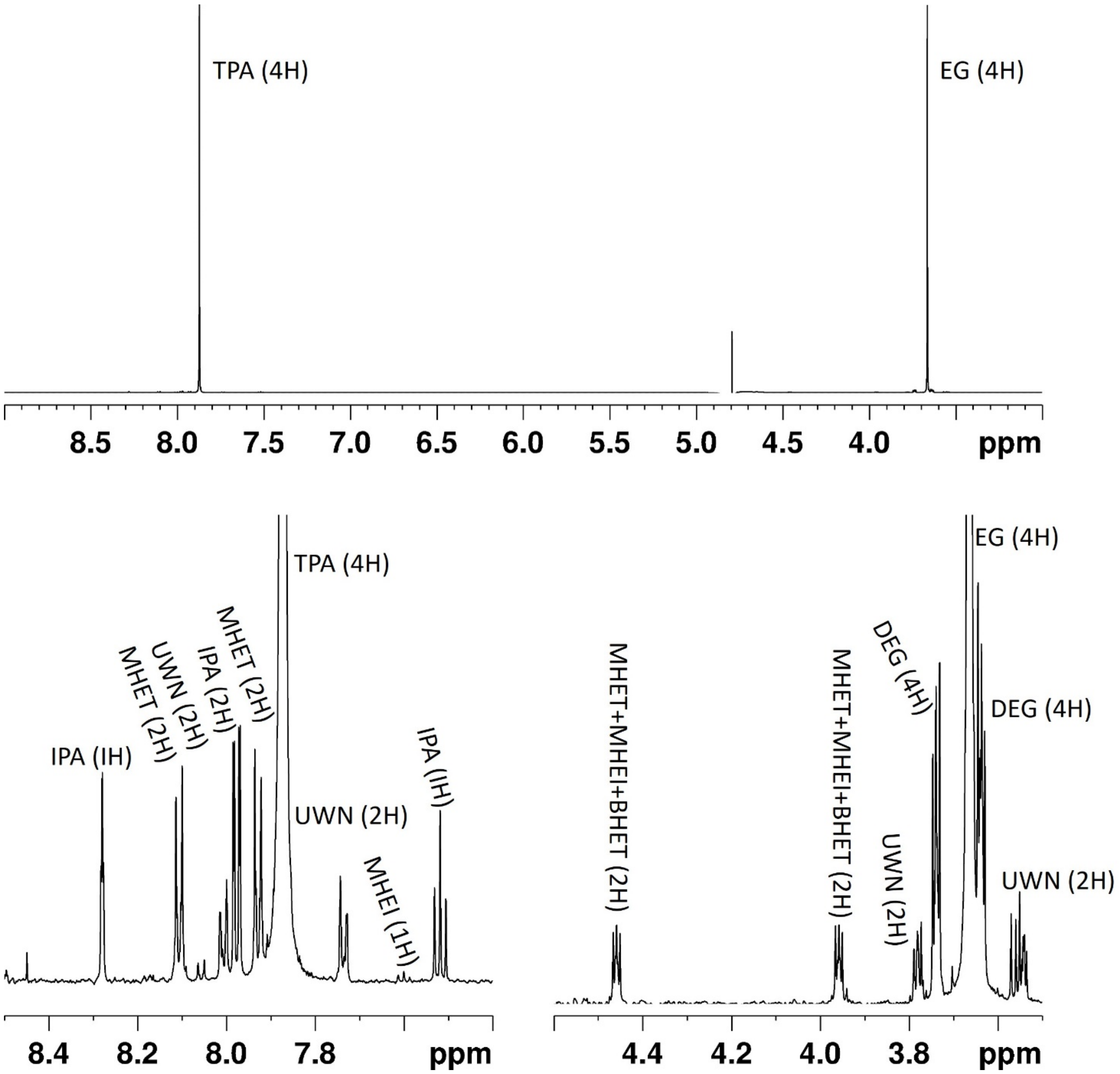

Fig. S11. DSC analysis of different PET materials

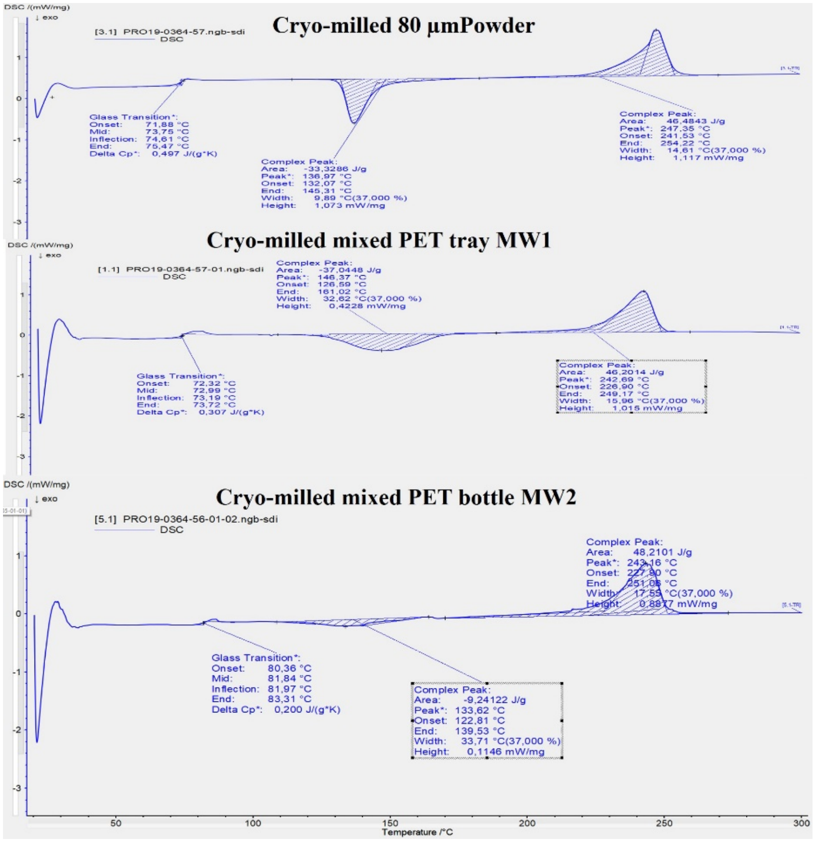

Amino acid sequences

>FastPETase

MQTNPYARGPNPTAASLEASAGPFTVRSFTVSRPSGYGAGTVYYPTNAGGTVGAIAIVPGY
TARQSSIKWWGPRLASHGFVVITIDTNSTLDQPESRSSQQMAALRQVASLNGTSSSPIYGKV
DTARMGVMGWSMGGGGSLISAANNPSLKAAAPQAPWHSSTNFSSVTVPTLIFACENDSIA
PVNSSALPIYDSMSQNAKQFLEIKGGSHSCANSGNSNQALIGKKGVAWMKRFMDNDTRYS
TFACENPNSTAVSDFRTANCSLELEHHHHHH

>LCC

MSNPYQRGPNPTRSALTADGPFSVATYTVSRLSVSGFGGGVIYYPTGTSLTFGGIAMSPGYT
ADASSLAWLGRRLASHGFVVLVINTNSRFDYPDSRASQLSAALNYLRTSSPSAVRARLDAN
RLAVAGHSMGGGGTLRIAEQNPSLKAAVPLTPWHTDKTFNTSVPVLIVGAEADTVAPVSQ

HAIPFYQNLPSTTPKVYVELDNASHFAPNSNNAAISVYTISWMKLWVDNDTRYRQFLCNV
NDPALSDFRTNNRHCQLEHHHHHH

>ICCG

MSNPYQRGPNPTRSALTADGPFSVATYTVSRLSVSGFGGGVIYYPTGTSLTFGGIAMSPGYT
ADASSLAWLGRRLASHGFVVLVINTNSRFDGPDSRASQLSAALNYLRTSSPSAVRARLDAN
RLAVAGHSMGGGGTLRIAEQNPSLKAAVPLTPWHTDKTFNTSVPVLIVGAEADTVAPVSQ
HAIPFYQNLPSTTPKVYVELCNASHIAPNSNNAAISVYTISWMKLWVDNDTRYRQFLCNVN
DPALCDFRTNNRHCQLEHHHHHH

References

[1] S. Fritzsche, H. Hübner, M. Oldiges, and K. Castiglione, Comparative evaluation of the extracellular production of a polyethylene terephthalate degrading cutinase by *Corynebacterium glutamicum* and leaky *Escherichia coli* in batch and fed-batch processes. *Microbial cell factories*, 23 (2024) 274. https://doi.org/10.1186/s12934-024-02547-2

[2] Y.V. Soong, U. Abid, A.C. Chang, C. Ayafor, A. Patel, J. Qin, J. Xu, C. Lawton, H.W. Wong, M.J. Sobkowicz, and D. Xie, Enzyme selection, optimization, and production toward biodegradation of post-consumer poly(ethylene terephthalate) at scale. Biotechnol J, 18 (2023) e2300119. https://doi.org/10.1002/biot.202300119

[3] G. Arnal, J. Anglade, S. Gavalda et al., Assessment of Four Engineered PET Degrading Enzymes Considering Large-Scale Industrial Applications. ACS Catal. 26, (2023) 13156-13166. 10.1021/acscatal.3c02922. https://doi.org/10.1021/acscatal.3c02922

[4] C.C. Chen, X. Li, J. Min, Z. Zeng, Z. Ning, H. He et al., Complete decomposition of poly (ethylene terephthalate) by crude PET hydrolytic enzyme produced in *Pichia pastoris*. Chem. Eng. J. 48, (2024) 148418. https://doi.org/10.1016/j.cej.2023.148418
